# Diaphragm thickening fraction to predict weaning—a prospective exploratory study

**DOI:** 10.1186/s40560-017-0258-4

**Published:** 2017-11-13

**Authors:** Sujay Samanta, Ratender Kumar Singh, Arvind K. Baronia, Banani Poddar, Afzal Azim, Mohan Gurjar

**Affiliations:** Department of Critical Care Medicine, Sanjay Gandhi Post Graduate Institute of Medical Sciences (SGPGIMS), Raebareli Road, Lucknow, Uttar Pradesh 226014 India

**Keywords:** Weaning, Diaphragm ultrasound, Diaphragm thickening fraction

## Abstract

**Background:**

Diaphragm ultrasound (DUS) is a well-established point of care modality for assessment of dimensional and functional aspects of the diaphragm. Amongst various measures, diaphragmatic thickening fraction (DT*f*) is more comprehensive. However, there is still uncertainty about its capability to predict weaning from mechanical ventilation (MV). The present prospective observational exploratory study assessed the diaphragm at variable negative pressure triggers (NPTs) with US to predict weaning in ICU patients.

**Methods:**

Adult ICU patients about to receive their first T-piece were included in the study. Linear and curvilinear US probes were used to measure right side diaphragm characteristics first at pressure support ventilation (PSV) of 8 cmH2O with positive end expiratory pressure (PEEP) of 5 cmH2O against NPTs of 2, 4, and 6 cmH2O and then later during their first T-piece. The measured variables were then categorized into simple weaning (SW) and complicated weaning (CW) groups and their outcomes analyzed.

**Results:**

Sixty-four (M:F, 40:24) medical (55/64, 86%) patients were included in the study. Sepsis of lung origin (65.5%) was the dominant reason for MV. There were 33 and 31 patients in the SW and CW groups, respectively. DT*f* predicts SW with a cutoff ≥ 25.5, 26.5, 25.5, and 24.5 for 2, 4, and 6 NPTs and T-piece, respectively, with ≥ 0.90 ROC AUC. At NPT of 2, DT*f* had the highest sensitivity of 97% and specificity of 81% [ROC AUC (CI), 0.91 (0.84–0.99); *p* < 0.001].

**Conclusions:**

DT*f* may successfully predict SW and also help identify patients ready to wean prior to a T-piece trial.

## Background

Weaning from mechanical ventilation (MV) is one of the major challenges faced by intensivists. Premature [1, 2] and delayed [3, 4] weaning are both detrimental in patients admitted in the intensive care unit (ICU). Weaning consumes approximately 40% time of ventilation [5]. While majority weaning is simple, difficult weaning is encountered in 20–25% of patients [6]. The diaphragm, the main inspiratory muscle, is affected by multiple factors in critical illness [7, 8], including disuse atrophy as a result of MV itself [9–12]. Diaphragm dysfunction also results in prolonged MV, weaning failure [13, 14], and increased mortality [15].

In 2007, the International Task Force of Respiratory and Critical Care Societies categorized weaning into simple, difficult, and prolonged [16]. Later in 2010, the incidence and outcomes of these new weaning categories were further studied [17]. In spite of subjective and objective extubation and weaning criteria, predicting a successful outcome is still difficult. Although several traditional tools to predict successful outcomes exist, their precision and accuracy are variable [18–20]. Diaphragm ultrasound (DUS) is a well-established point of care modality for assessment of dimensional and functional aspects of the diaphragm [14, 21]. Diaphragmatic thickening fraction (DT*f* (%)) reflects the magnitude of diaphragmatic effort and may predict successful weaning [22, 23].

We proposed to confirm the utility of DUS to assess muscle function in response to a maximal volitional inspiratory effort. In order to test the hypothesis that DUS-based measurements can successfully predict weaning, we conducted the present prospective exploratory study in adult critically ill ICU patients at variable negative pressure triggers (NPTs) both prior and during a T-piece trial. We in our present study also attempted to explore DUS-based parameters in the above-mentioned weaning categories.

## Methods

### Ethics and consent

After prior approval from the ethics committee (Sanjay Gandhi Post Graduate Institute of Medical Sciences, Lucknow, UP, India) and obtaining patient’s written informed consent, we conducted the present prospective exploratory study. The study period was from January 2015 to June 2016. A 12-bed closed, medical, surgical, adult, and pediatric ICU of a tertiary care referral hospital and academic institute in north of India was used for this purpose. The clinical management of patients was at the discretion of the ICU treating team in accordance with the contemporary best ICU practices. No interventions or therapy was modified based on the study findings.

### Inclusion criteria

Patients aged ≥ 18 years, admitted to ICU and receiving MV longer than 24 h and about to be subjected to their first T-piece after satisfying conventional criteria for ready to wean from ventilator, were enrolled in the study. DUS examinations were performed initially at pressure support ventilation (PSV) with variable NPTs, and then 6–12 h later during the first T-piece trial.

### Exclusion criteria

Patients aged < 18 years, ventilated for less than 24 h, with preexisting diaphragm disease, increased intra-abdominal pressure, any breach in skin preventing DUS examinations in subcostal area, phrenic nerve palsy, and refusal of consent were excluded from study. Patients who deteriorated with application of PSV at NPTs or during the T-piece were also excluded.

### Study protocol

Patients on MV received their first T-piece when they were afebrile, alert, cooperative, and hemodynamically stable without vasopressor support and PaO_2_/FiO_2_ ratio > 200 was achievable at FiO_2_ < 0.5 with positive end expiratory pressure (PEEP) ≤ 5 cmH2O and respiratory rate of < 30 breaths per minute. The patients who were considered ready to wean from MV as per the above indices were included in the assessment of increasing ventilatory burden by subjecting them to non-randomized NPTs of 2, 4, and 6 during PSV of 8 cmH2O with PEEP 5 cmH2O. A period of 30 min of PSV without NPT was mandated to prevent exhaustion from burden of the test. Patients who successfully tolerated the variable NPT trial subsequently received their first T-piece trial after 6–12 h to prevent influence of any burden of test on the outcome of T-piece. Both PSV at NPTs and the T-piece trials were performed in semi-recumbent position. Decisions about tolerability of NPTs, T-piece, extubation, repeat T-piece, and or tracheostomy were as per the clinical judgment of the physician in-charge of the patient and were not in any way based on DUS measurements.

### Diaphragm ultrasound

DUS measurements were performed on the right subcostal side using both brightness (B) and motion (M) mode.

#### Ultrasound machine and probe

High-resolution linear and curvilinear US probes of 10 and 3.5–5 MHz (FUJIFILM SonoSite, Inc.) were used to measure the diaphragm thickness (DT) and diaphragmatic excursion [amplitude (AMP)] respectively using both B and M modes.

#### Probe placement

Both the amplitude and speed of contraction were assessed by placing the curvilinear probe on the right subcostal margin between the mid-clavicular and anterior axillary line allowing placement of the M mode line parallel to the excursion of the diaphragm. DT was measured in the zone of apposition of the diaphragm and rib cage in the mid-axillary line between the eighth and tenth intercostal space. The right-sided DUS measurements were used because of their reproducibility and feasibility in MV patients [21].

#### Measurements

DT [at end of inspiration (*i*) and expiration (*e*)], AMP [centimeters (cm)], and speed of contraction [SP*cont* (cm/s)] were measured. The DT*f* (%) was calculated as the difference between DT*i* and DT*e* divided by DT*e ×* 100. These measurements were performed by a single intensivist experienced in performing DUS. In order to minimize intra-observer variability to less than 10% and establish reproducibility, an average of three readings measured in at least three sessions each lasting 10–15 min was ensured.

#### Inspiratory effort capacity

Within 6–12 h preceding the first T-piece, each patient was subjected to NPTs of 2, 4, and 6 cmH_2_O at PSV of 8 and 5 cmH2O PEEP for a minimum period of 20 min each to achieve a steady state. The measurements were recorded at the end of the 20th minute. Cooperative patient was instructed to perform breathing to total lung capacity (TLC) and then to exhale to residual volume (RV). DUS measurements at TLC and RV were then recorded. These points were considered as surrogates of end-inspiration and end-expiration respectively [22]. Several images of the diaphragm were captured and stored, including at least three at the point of maximum thickening at TLC and at least three at minimum thickening at RV. Diaphragm measurements were taken at PSV at three different NPTs and during the period of the first T-piece and at TLC and RV. Between each change over to a higher NPT, a rest period of 30 min on previous ventilatory support was mandatory to prevent exhaustion. The protocol was also interrupted for 30 min with increased pressure support after each trigger if signs of respiratory distress like respiratory rate > 35 breaths/min, SpO2 < 90%, heart rate > 140 beats/min, variation of > 30% from baseline, systolic blood pressure > 180 or < 90 mmHg, diaphoresis, or anxiety occurred. The time gap of 6–12 h between NPT trials and T-piece was incorporated to provide enough rest between the two procedures. SERVO-i-Maquet ventilator was used for mechanical ventilation of all patients included in the study.

### Definitions

Patients were categorized based on the following weaning classification [16].

#### Simple weaning

Patients who proceeded from initiation of weaning to successful extubation on their first SBT without any difficulty were categorized as simple weaning (SW).

#### Difficult weaning

Patients who failed initial weaning and required up to three SBTs or as long as 7 days from the first SBT to achieve successful weaning were categorized as having difficult weaning.

#### Prolonged weaning

Patients who failed at least three weaning attempts or required 7 days of weaning after the first SBT were said to have prolonged weaning.

#### Weaning failure

It was defined as resumption of ventilatory support within 48 h of liberation from MV.

#### Complicated weaning

We grouped all patients with difficult, prolonged, and failed weaning together as complicated weaning (CW).

### Data collection

Demographic (age, gender, category of patient, care received prior to present admission, source of admission, type of illness, coexisting illness, and source of sepsis), severity [Acute Physiologic and Chronic Health Evaluation (APACHE-II) and Sequential Organ Dysfunction Assessment (SOFA)] scores, organ failure at admission, indication for intubation, ventilation-related characteristics like tracheostomy, spontaneous breathing trials (SBTs), time before initiation of T-piece, length of MV, and ICU stay, along with DUS-based parameters of thickness, amplitude, thickening fraction, and outcomes relating to SW and CW and 28-day survival, were all recorded.

### Sample size and statistical analysis

#### Sample size

Sample size was calculated assuming simple weaning proportion of 0.5 and 25% relative error of the proportion at two-sided 95% confidence interval (CI). Finally, a minimum sample size of 62 was calculated for the study. Sample size was calculated using software power analysis and sample size (PASS version 8).

#### Statistical analysis

Normality of continuous data was tested using Shapiro-Wilk test. Non-normal, continuous data was expressed as median (interquartile range), while categorical data was expressed as frequency and percentage. Mann-Whitney *U* test was used to compare the medians between SW and CW. Kruskal-Wallis test was used for comparison of continuous variables between more than two groups. Chi-square test was used to compare the proportions/test the association between groups. For repeated observations over variable NPTs, Friedman analysis of variance (ANOVA) was used to estimate the significance. If in Friedman ANOVA the *p* value was observed to be significant, then the difference in medians between individual groups was further assessed using the Wilcoxon signed rank test. A two-tailed *p* value of < 0.05 was considered statistically significant. IBM, SPSS version 23 (SPSS Inc., Chicago, IL, USA), was used for statistical analysis.

## Results

Sixty-four patients, 40 (62.5%) males, were included in the study. Baseline characteristics of the studied population were as depicted in Table 1. Approximately, 86% of patients were with medical illness. Prior to present ICU admission, nearly 73 and 48% received ICU and MV support, respectively. Nearly 45% of the studied patients had been transferred from ICUs of other hospitals. Sepsis was the predominant (17/64, ~ 27%) reason for admission, with nearly 66% of respiratory origin. Nearly 58% of patients had no coexisting illness. There were 33 and 31 patients in SW and CW group, respectively. The groups were not significantly different, except for the type of illness (*p*, 0.01) (Table 1). Amongst the CW group, there were 16, 10, and 5 patients with difficult, prolonged, and failed weaning, respectively. Their baseline characteristics were also comparable with SW (Table not shown).

**Table 1:** Baseline characteristics of patients with different weaning outcomes

Variables	All weaning (*N* = 64)	Simple weaning (*n* = 33)	Complicated weaning (*n* = 31)	*p* value
Age, years	37 (22–56)	39 (21–54)	37 (22–58)	0.37
Gender, male, *n* (%)	40 (62.5)	22 (66.7)	18 (58.1)	0.60
Category of patients, *n* (%)				0.29
Medical	55 (85.9)	30 (90.9)	25 (80.6)	
Surgical	9 (14.1)	3 (9.1)	6 (19.3)	
Care received prior to admission, *n* (%)				
Intensive care	47 (73.4)	24 (72.7)	23 (74.2)	1
Mechanical ventilation	31 (48.4)	13 (39.4)	18 (58.1)	0.21
Source of admission, *n* (%)				0.05
Emergency	12 (18.8)	5 (15.1)	7 (22.6)	
Intra-hospital ICU	14 (21.9)	11 (33.3)	3 (9.7)	
Inter-hospital ICU	29 (45.3)	11 (33.3)	18 (58.1)	
Intra-hospital ward	9 (14.1)	6 (18.2)	3 (9.7)	
Type of illness, *n* (%)				
Sepsis	17 (26.6)	11 (33.3)	6 (19.3)	0.01
Neurological	16 (25)	3 (9.1)	13 (41.9)	
Cardiovascular	3 (4.7)	3 (9.1)	0	
Gastrointestinal	3 (4.7)	0	3 (9.7)	
Hepatic	4 (6.3)	2 (6.1)	2 (6.4)	
Respiratory	3 (4.7)	2 (6.1)	1 (3.2)	
SAP	4 (6.3)	1 (3.0)	3 (9.7)	
Tropical	7 (10.9)	5 (15.1)	2 (6.4)	
Others	7 (10.9)	6 (9.1)	1 (3.2)	
Coexisting illness, *n* (%)				0.66
CKD	3 (4.7)	3 (9.1)	0	
COPD	3 (4.7)	1 (3.0)	2 (6.4)	
Hypertension	6 (9.4)	2 (6.1)	4 (12.9)	
Hypertension, diabetes mellitus	7 (10.9)	3 (9.1)	4 (12.9)	
Hypothyroidism	1 (1.6)	0	1 (3.2)	
Multiple (≥ 3)	3 (4.7)	2 (6.1)	1 (3.2)	
None	37 (57.8)	20 (60.6)	17 (54.8)	
Other	4 (6.3)	2 (6.1)	2 (6.4)	
Source of sepsis, *n* (%)				0.60
Intra-abdominal	8 (12.5)	3 (9.1)	5 (16.1)	
Central nervous system	8 (12.5)	5 (15.1)	3 (9.7)	
Hepatic	1 (1.6)	1 (3.0)	0	
Respiratory	42 (65.6)	20 (60.6)	22 (71.0)	
None	1 (1.6)	1 (3.0)	0	
Unknown	4 (6.3)	3 (9.1)	1 (3.2)	

Data is expressed in median (interquartile range), unless specified. Test for statistical significance (*p* value) used is Pearson chi-square for categorical data and Mann-Whitney *U* test for continuous measurements

*Abbreviations*: *IQR* interquartile range, *ICU* intensive care unit

The attributes of severity, MV, and outcomes were as depicted in Table 2. APACHE-II and SOFA scores were comparable. Nearly 95% (61/64) of patients had two or more organ failures at admission, and they differed significantly between SW and CW (*p*, 0.04). Nearly 37% (24/64) required tracheostomy (SW, 1 vs. CW, 23; *p* < 0.001) during ICU stay. Significantly delayed [CW, 13 (8–22) day vs. 6 (4–8.5) day in SW; *p* < 0.001)] and failed first T-piece [CW, 20 (64.5%) vs. Nil in SW; *p* < 0.001] along with prolonged MV [CW, 22 (14–28) vs. 6 (5–9) in SW; *p* < 0.001] and ICU stay [CW, 28 (15–35) days vs. 8 (7–14.5) days in SW; *p* < 0.001] were observed in CW relative to SW (Table 2). Similar significance (*p* < 0.001) for these characteristics were also observed when comparing SW with difficult, prolonged, and failed weaning (Table not shown). Only one patient in the SW group got re-intubated and was later tracheostomized during the follow-up period of 28 days. All patients in CW group (*n*, 31) failed the first T-piece trial, and 23 (23/31, 74%) were tracheostomized. The remaining eight patients were extubated following more than one T-piece trial in the follow-up period. Post extubation non-invasive ventilation was used in eight patients in the CW group.

**Table 2:** Severity, ventilation, and outcome-related characteristics in weaning groups

Variables	All weaning (*N* = 64)	Simple weaning (*n* = 33)	Complicated weaning (*n* = 31)	*p* value
Severity scores at admission				
APACHE-II	12 (10–16)	12 (10–15)	14 (10–16)	0.47
SOFA	8 (6–10)	8 (6–10)	8 (6–10)	0.74
No of organ failure at admission, *n* (%)				0.04
1	3 (4.7)	0	3 (9.7)	
2	29 (45.3)	18 (54.5)	11 (35.5)	
3	23 (35.9)	13 (39.4)	10 (32.2)	
4	9 (14.1)	2 (6.1)	7 (22.6)	
Indication for intubation, *n* (%)				0.44
Central nervous system	20 (31.3)	9 (27.3)	11 (35.5)	
Respiratory	35 (54.7)	20 (60.6)	15 (48.4)	
Combination	2 (3.1)	0	2 (6.4)	
Others	7 (10.9)	4 (12.1)	3 (9.7)	
Onset of illness requiring ICU admission, days	7 (5–11.7)	7 (5–12)	7 (6–10)	0.79
Tracheostomy, *n* (%)	24 (37.5)	1 (3.0)	23 (74.2)	< 0.001
1st T-piece failure, *n* (%)	20 (31.3)	0	20 (64.5)	< 0.001
Time to T-piece, days	8 (5–14)	6 (4–8.5)	13 (8–22)	< 0.001
MLV, days	12.5 (6–21.7)	6 (5–9)	22 (14–28)	< 0.001
LOS-ICU, days	14.5 (8–28)	8 (7–14.5)	28 (15–35)	< 0.001
Survival at 28 days, *n* (%)	60 (93.8)	33 (100)	27 (87.1)	0.05
Final outcome, *n* (%)				0.07
Death	4 (6.3)	0	4 (12.9)	
Discharged to home	39 (60.9)	20 (60.6)	19 (61.3)	
Transferred to ward	21 (32.8)	13 (39.4)	8 (25.8)	

Data is expressed in median (interquartile range), unless specified. Test for statistical significance (*p* value) used is Pearson chi-square for categorical data and Mann-Whitney *U* test for continuous measurements

*Abbreviations*: *APACHE* Acute Physiological and Chronic Health Evaluation, *SOFA* Sequential Organ Failure Assessment, *ICU* intensive care unit, *MLV* mechanical lung ventilation, *LOS* length of stay

While 100% of patients in SW, difficult and prolonged weaning, survived 28 days, one of five (20%) in the failed weaning died within this period (*p* < 0.001) (Table not shown). The 28-day survival and final outcomes were comparable (Table 2). However, the four patients with CW who finally died were also the ones who had failed weaning and three of them died beyond 28 days of ICU stay.

The measurements of diaphragm (DT*i*, DT*e*, DT*f*, AMP, and SP*cont*) at NPTs of 2, 4, and 6 and during first T-piece for SW and CW were as depicted in Table 3. Also depicted in the same table were inter- and intra-differences between groups. DT*i* exceeded DT*e* at all NPTs. The intergroup variability between SW and CW was statistically significant for DT*i*, DT*f*, AMP, and SP*cont*, at variable NPTs and T-piece (for each, *p* < 0.001). Similar comparison of SW with difficult, prolonged, and failed weaning patients was also significant (*p* < 0.001) (Table not shown). The intra-group variability at different triggers and T-piece as assessed by Friedman ANOVA was statistically significant (*p* ≤ 0.001) for all the measured diaphragm parameters except in the CW for DT*f* (*p*, 0.34). Similar results of significance were also observed when SW was compared with difficult and prolonged weaning (*p* ≤ 0.001), except that patients with failed weaning had a somewhat lower significance (*p* < 0.05) (Table not shown). The ∆2–4, ∆4–6, and ∆2–6 variables for SW and CW were as depicted in Table 3. For most, the significance was ≤ 0.001, except for 2, 3, and 4 variables in ∆2–4, ∆4–6, and ∆2–6 respectively, wherein it was comparable. However, the ∆ variability (Table 3) increased when patients in SW were compared with difficult, prolonged, and failed weaning (Table not shown).

**Table 3:** Differences between groups at variable negative pressure

Diaphragm parameters	Weaning groups (*N* = 64)	Negative pressure trigger (*N* = 64)							
Median (IQR)	Simple (*n* = 33) Complicated (*n* = 31)	2	4	6	T-piece	*p* ^##^	*p* ^###^		
							∆2–4	∆4–6	∆2–6
DT*i,* mm	Simple	2.2 (2.1–2.3)	2.2 (2.2–2.3)	2.2 (2.2–2.3)	2.2 (2.2–2.2)	< 0.001*	< 0.001*	< 0.001*	<0.001*
	Complicated	2.0 (2.0–2.1)	2.1 (2–2.2)	2.0 (2.0–2.2)	2.0 (2.0–2.2)	< 0.001*	< 0.001*	< 0.001*	0.004
	*p* ^#^	< 0.001	< 0.001	< 0.001	< 0.001				
DT*e,* mm	Simple	1.7 (1.6–1.7)	1.7 (1.7–1.8)	1.7 (1.7–1.8)	1.7 (1.6–1.8)	< 0.001*	< 0.001*	0.82	<0.001*
	Complicated	1.6 (1.5–1.7)	1.7 (1.6–1.8)	1.6 (1.6–1.8)	1.7 (1.6–1.8)	0.001	< 0.001*	0.03	0.006
	*p* ^#^	0.22	0.31	0.10	0.16				
DT*f*, %	Simple	31 (28–32)	31 (28–35)	29 (26–32)	29 (26–33)	0.010	0.40	0.001	0.05
	Complicated	22 (17–24)	22 (18–27)	23 (18–25)	21 (17–25)	0.34	0.94	0.28	0.50
	*p* ^#^	< 0.001	< 0.001	< 0.001	< 0.001				
AMP, cm	Simple	1.3 (1.3–1.5)	1.3 (1.3–1.5)	1.3 (1.2–1.4)	1.3 (1.2–1.4)	< 0.001*	< 0.001*	< 0.001*	0.49
	Complicated	1.2 (1.1–1.2)	1.2 (1.1–1.3)	1.2 (1.1–1.2)	1.2 (1.1–1.2)	< 0.001*	< 0.001*	0.01	<0.001*
	*p* ^#^	< 0.001	< 0.001	< 0.001	< 0.001				
SP*cont*, cm/s	Simple	1.3 (1.3–1.3)	1.3 (1.3–1.4)	1.3 (1.3–1.4)	1.3 (1.2–1.3)	< 0.001*	0.001	< 0.001*	0.36
	Complicated	1.2 (1.1–1.2)	1.2 (1.1–1.3)	1.2 (1.1–1.3)	1.2 (1.1–1.3)	0.001	< 0.001*	0.97	<0.001*
	*p* ^#^	< 0.001	< 0.001	< 0.001	< 0.001				

Note: Tests of statistical significance used for *p* values: *p* ^#^, Mann-Whitney *U* test; *p* ^##^, Friedman ANOVA test; *p* ^###^, Wilcoxon signed rank test; p*, < 0.001 highly significant

*Abbreviations*: *DTi* thickness of diaphragm during inspiration, *DTe* thickness of diaphragm during expiration, *TP* T-piece, *DTf* diaphragm thickening fraction, *AMP* amplitude of diaphragm, *SPcont* speed of contraction of diaphragm

The sensitivity and specificity of various diaphragm measurements to predict SW was analyzed using the receiver operative characteristics (Table 4). With a cutoff at or above 25.5, 26.5, 25.5, and 24.5 for 2, 4, and 6 NPTs and T-piece, respectively, the DT*f* had a ROC AUC of ≥ 0.90. At NPT of 2, DT*f* had the highest sensitivity of 97%, albeit 81% specificity [ROC AUC, 0.91 (0.84–0.99); *p* < 0.001] compared to AMP and SP*cont.*

**Table 4:** Prediction of simple weaning

Diaphragm parameter	Cutoff	ROC of AUC (95% CI)	Sensitivity (%)	Specificity (%)	*p* value
DT*f,* % NPT					
2	≥ 25.5	0.91 (0.84–0.99)	97	81	< 0.001
4	≥ 26.5	0.93 (0.87–0.99)	87	75	< 0.001
6	≥ 25.5	0.91 (0.84–0.99)	90	88	< 0.001
T-piece	≥ 24.5	0.90 (0.82–0.97)	87	75	< 0.001
AMP, inspiration, cm					
2	≥ 1.21	0.87 (0.78–0.96)	87	75	< 0.001
4	≥ 1.27	0.81 (0.71–0.92)	81	81	< 0.001
6	≥ 1.24	0.77 (0.66–0.88)	84	59	< 0.001
T-piece	≥ 1.20	0.76 (0.65–0.88)	72	55	< 0.001
SP*cont*, cm/s					
2	≥ 1.24	0.94 (0.88–0.99)	84	91	< 0.001
4	≥ 1.27	0.94 (0.89–0.99)	93	78	< 0.001
6	≥ 1.24	0.89 (0.81–0.96)	78	71	< 0.001
T-piece	≥ 1.23	0.86 (0.77–0.94)	87	68	< 0.001

*Abbreviations*: *ROC* receiver operative characteristics, *AUC* area under curve, *CI* confidence interval, DTf diaphragm thickening fraction, *AMP* amplitude of diaphragm, *SPcont* speed of contraction of diaphragm

## Discussion

DUS is an acknowledged investigative tool for assessment of diaphragm in critically ill patients. The present prospective study utilized DUS measurements at variable inspiratory efforts to predict successful weaning. The main findings of our study were as follows: (1) DT*f* predicts simple weaning; (2) DUS parameters at variable NPTs can identify patients ready to wean; and (3) DUS can help analyze patients with complicated weaning.

### DT*f* predicts simple weaning

In recent years, several DUS-based measurements and some derived parameters have been validated for predicting weaning in critically ill patients [13, 14, 21–23]. Similar to majority of previous studies [21, 24, 25], we too assessed the more feasible and highly reproducible right hemi-diaphragm via DUS. DT*i*, DT*f*, AMP, and SP*cont* were all significantly higher in SW compared to CW (Table 3) or difficult, prolonged, and failed weaning (Table not shown), both at variable NPTs and during T-piece (*p* < 0.001). These parameters were also relatively better at different NPTs than during T-piece for predicting SW. The DT*f* cutoff ≥ 25.5% with AUC of 0.91 had sensitivity and specificity of 97 and 81% respectively at NPT of 2 for predicting SW. This sensitivity was higher than the AMP (cutoff ≥ 1.21 cm) and SP*cont* (cutoff ≥ 1.24 cm/s) at same NPT, desirable to predict SW. Variable DT*f* cutoffs have been used previously. DiNino et al., in 2014, studied DUS in 63 patients before extubation, during SBT or pressure support trial [23]. They suggested that a threshold DT*f* of greater or equal to 30%, with a positive predictive value (PPV) and negative predictive value (NPV) of 91 and 63%, respectively, for extubation success, performed similarly during SBT or pressure support ventilation. Similarly, Ferrari et al., in 46 patients of repeated weaning failure, suggested that a cutoff DT*f* greater or equal to 36%, during SBT in tracheostomized patients, was associated with a PPV and NPV of 92 and 75%, respectively, for successful or failed weaning at 48 h [22]. By comparison, rapid shallow breathing index (RSBI) < 105 had sensitivity, specificity, PPV, and NPV of 93, 88, 93, and 88%, respectively, for determining successful SBT. The plausible explanation of a lower DT*f* threshold in our study is due to differences in methodology, variable inspiratory efforts, patient population, and severity of illness at ICU admission, metabolic conditions, and duration of MV. Several studies report superiority of DT*f* over diaphragm excursions as a marker of diaphragm function [26, 27]. However, we studied predictability of weaning via DUS at variable inspiratory efforts and observed a higher sensitivity and comparable AUC for DT*f* to predict SW.

### DUS parameters at variable NPTs can identify patients ready to wean

The ROC AUC, sensitivity, and specificity during T-piece for DT*f*, AMP, and SP*cont* were lower as compared to NPTs at comparable cutoffs (Table 4). DUS measurements at NPT of 2 were observed to be more favorable for predicting SW compared to T-piece. Both, DT*f* and AMP, showed higher or comparable sensitivity and specificity at NPT of 2 relative to NPT of 6. Hence, SW prediction can be performed prior to T-piece and at lower NPTs. No previous studies have reported this.

### DUS can help analyze patients with complicated weaning

Diaphragm excursion [13], twitch tracheal pressure [15], and trans-diaphragmatic twitch pressure [26, 27] have all been used to quantify diaphragm dysfunction. These studies have reported increased mortality and morbidity associated with diaphragm dysfunction. However, we categorized our patients into SW and CW*.* All DUS parameters were significantly lower in the CW group, and these patients also had delayed and failed SBT and prolonged MV and length of ICU stay. Similar outcomes were observed in the difficult, prolonged, and failed weaning patients. These were similar to findings of earlier study [17]. Only patients with failed weaning died.

### Limitations

Several limitations of our study were as follows: (1) single-center study with a small sample size; (2) DT*f* cutoff not validated; (3) illness- and severity-specific variability not ascertained due to a small sample size; (4) minimal differences between measurements altered the weaning categorization; (5) small number of patients in difficult, prolonged, and failed weaning may have overestimated the differences; (6) trigger sensitivity not randomized; (7) due to small sample size, independent trigger sensitivity for each patient group could not be individually tested; (8) workload increase contributed by the burden of test and its impact on T-piece outcome could not be clearly ascertained; ideally, these two observations should have been done separately to avoid influence; (9) rest period of 30 min between variable triggers may have been insufficient to relieve the fatigue imposed; (10) DUS-based measurements were not compared against traditional weaning indices; (11) trends of DUS measurements overtime in CW group may have better correlated with outcomes; (12) inter-observer variability not assessed; (13) due to non-availability of ventilator-coupled USG machine, only cooperative patients could be included in the study; (14) factors affecting the DT*f*, their impact on weaning, and how they could be modified to optimize weaning were not studied.

Despite the abovementioned shortcomings, our study is a humble exploratory attempt to use DUS measurements to identify patients with SW even before their first T-piece trial. DT*f*, AMP, and SP*cont* with cutoffs ≥ 25.5%, ≥ 1.2 cm, and ≥ 1.24 cm/s, respectively, at NPT of 2 may help to not only determine which patient will safely endure the T-piece but also predict a successful T-piece trial. These measures may also help to further analyze patients with CW. Furthermore, DT*f* values could also be of use to optimize CW patients for further extubation trials.

## Conclusion

Ultrasound-based diaphragm measurements at variable inspiratory efforts can help identify patients safe and ready to wean even without enduring a T-piece. Amongst these parameters, DT*f*, apart from recognizing readiness to wean, can also predict simple weaning. However, a larger multicenter study is still required to validate the observed DT*f* cutoff in our study. Research on factors which affect the DT*f* and their modification to optimize weaning need to be further explored.

## Abbreviations

AMP: Amplitude
APACHE: Acute Physiology and Chronic Health Evaluation
CW: Complicated weaning
DT: Diaphragm thickening
DT*e*: Diaphragm thickening during expiration
DT*f*: Diaphragm thickening fraction
DT*i*: Diaphragm thickening during inspiration
DUS: Diaphragm ultrasound
ICU: Intensive care unit
MV: Mechanical ventilation
NPTs: Negative pressure triggers
PSV: Pressure support ventilation
RV: Residual volume
SOFA: Sequential Organ Failure Assessment
SP*cont*: Speed of contraction
SW: Simple weaning
TLC: Total lung capacity
